# Association between breakfast skipping and metabolic outcomes by sex, age, and work status stratification

**DOI:** 10.1186/s12986-020-00526-z

**Published:** 2021-01-07

**Authors:** Jun Heo, Won-Jun Choi, Seunghon Ham, Seong-Kyu Kang, Wanhyung Lee

**Affiliations:** 1 College of Medicine, Gachon University, Incheon, Republic of Korea; 2 Department of Occupational and Environmental Medicine, Gil Medical Center, Gachon University College of Medicine, 21, Namdong-daero 774 beon-gil, Namdong-gu, Incheon, 21565 Republic of Korea

**Keywords:** Breakfast skipping, Abnormal metabolic outcomes, Working conditions

## Abstract

**Background:**

The association between breakfast skipping and abnormal metabolic outcomes remains controversial. A comprehensive study with various stratified data is required.

**Objective:**

The aim of this study was to investigate the relationship between abnormal metabolic outcomes and breakfast skipping by sex, age, and work status stratification.

**Methods:**

We used data from the Korea National Health and Nutrition Examination Surveys from 2013 to 2018. A total of 21,193 (9022 men and 12,171 women) participants were included in the final analysis. The risk of metabolic outcomes linked to breakfast skipping was estimated using the negative binomial regression analysis by sex, work status, and age stratification.

**Results:**

A total of 11,952 (56.4%) participants consumed breakfast regularly. The prevalence of abnormal metabolic outcomes was higher among those with irregular breakfast consumption habits. Among young male workers, negative binomial regression analysis showed that irregular breakfast eaters had a higher risk of abnormal metabolic outcomes, after adjusting for covariates (odds ratio, 1.15; 95% confidence interval, 1.03–1.27).

**Conclusions:**

The risk of abnormal metabolic outcomes was significant in young men in the working population. Further studies are required to understand the association of specific working conditions (working hours or shift work) with breakfast intake status and the risk of metabolic diseases.

## Introduction

Breakfast is the most important meal of the day because it helps the human body to start daily metabolism. The human body is regulated by circadian rhythms. Circadian rhythms are influenced by the light–dark cycle, as well as by food uptake, which is the metabolic signal. Inversely, circadian regulation of metabolic genes affects metabolic outcomes in the human body, which signifies that feeding time and the circadian clock are tightly intertwined [1]. Breakfast is important to jumpstart daily metabolism. A randomized clinical trial showed that breakfast skipping adversely affected circadian gene expression and correlated with increased postprandial glycemic response [2]. The irregular consumption of breakfast can induce various health problems.

Many studies have reported the association between breakfast skipping and health problems. A large, prospective study conducted in the US on middle-aged and older male health professionals in the US confirmed that eating breakfast was associated with a significantly lower risk of coronary heart disease [3]. Some studies found that individuals who skipped breakfast had higher rates of mortality [4], higher serum cholesterol levels [5], and frequent health-compromising behaviors [6], compared with regular breakfast eaters. In addition, other studies have reported that breakfast intake has many beneficial effects such as improved satiety, reduced incidence of food cravings [7] and improved cognitive function and academic performance [8].

Breakfast skipping has a significant impact on body weight and metabolic outcomes. The relationship between breakfast skipping and high body mass index (BMI) values has been widely reported in adolescent populations in Europe [9], Hong Kong [10], and Fiji (girls) [11]. Similar associations were reported in the adult [12], middle-aged adult [13], and elderly [14] populations. Many studies have reported the association between metabolic outcomes and breakfast skipping; however, more evidence is required. The strength of the association between breakfast habituation and metabolic outcomes varies according to age group, sex, and ethnicity. For instance, a cross-sectional study on 5316 American young adults showed that regular breakfast eaters were less likely to have elevated low-density lipoprotein cholesterol (LDL-C) levels, high blood pressure, and reduced serum high-density lipoprotein cholesterol (HDL-C) levels [15]. In contrast, a study on 415 Korean adults confirmed that regular breakfast intake was associated with elevated triglyceride (TG) levels [16]. Thus, studies on the association between breakfast skipping and metabolic syndrome remain conflicting, warranting further studies on this subject.

This study was undertaken to (1) identify the relationship between breakfast skipping and metabolic outcomes in the Korean adult population and (2) demonstrate, in detail, the effect of breakfast skipping on metabolic outcomes according to age group, sex, and work status.

## Methods

### Data and study participants

**Fig. 1:**
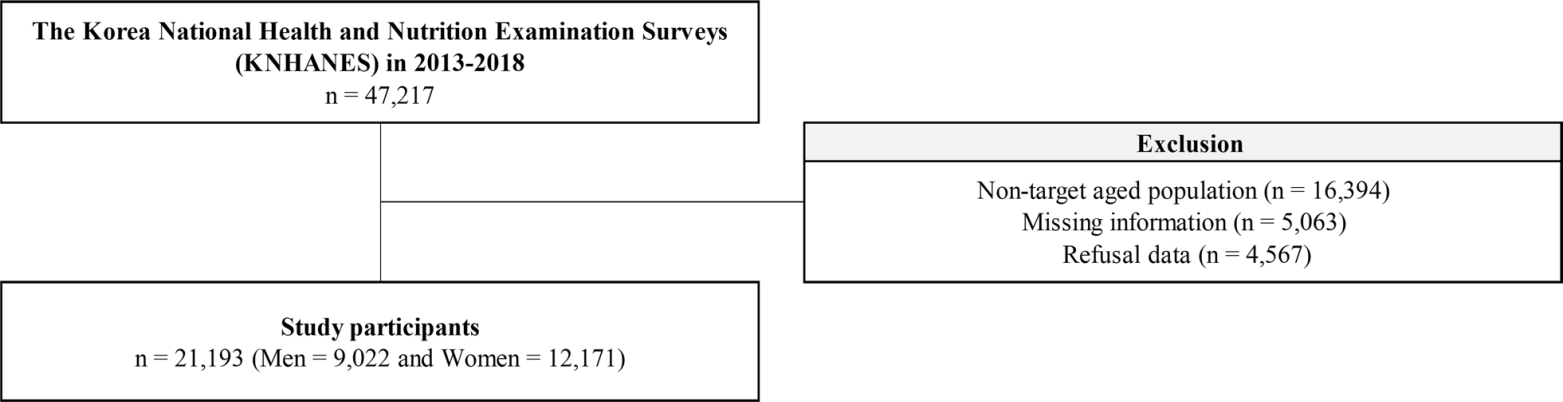
Schematic diagram depicting the study population

We used data from the Korea National Health and Nutrition Examination Surveys (KNHANES) from 2013 to 2018. KNHANES, which has been conducted every year since 1998 by the Korea Centers for Disease Control and Prevention (KCDC), is a series of nationally representative, population-based surveys on the health and nutritional status of Korean citizens [17]. The KNHANES database is publicly available on their website (http://knhanes.cdc.go.kr, available in Korean). More than 7000 participants were selected each year by the stratified random sampling method. Data were collected through interviews, blood tests, urine tests, and physical examinations in the examination vehicle. KNHANES was approved by the KCDC Institutional Review Board, and all participants provided written informed consent. The total number of participants in KNHANES from 2013 to 2018 was 47,217. We excluded the following participants: (1) those older than 59 years or less than 20 years of age (*n* = 16,394); (2) those who refused to answer questions regarding their work status (*n* = 5063); and (3) those with missing information for metabolic outcomes, frequency of breakfast consumption, education level, and household income (*n* = 4567). After all the exclusions, the final number of participants included in this analysis was 21,193 (9022 men and 12,171 women, Fig. 1).

### Status of breakfast consumption

The frequency of breakfast consumption was assessed using a self-administered questionnaire. Participants were asked to report the average number of breakfasts consumed per week in the past year; four categories were considered: 5–7/week, 3–4/week, 1–2/week, and 0/week. We then categorized the participants into two groups: (1) regular breakfast eaters (those who ate breakfast almost every day; 5–7/week category) and (2) irregular breakfast eaters (those who ate breakfast rarely or never; 3–4/week, 1–2/week, and 0/week categories).

### Measurement of metabolic outcomes

Metabolic outcomes included central obesity, raised blood pressure, raised fasting serum glucose, increased TG, and decreased HDL-C levels from definition of metabolic syndrome according to the National Cholesterol Education Program Adult Treatment Panel III (NCEP-ATP III) criteria [18]. Abnormal metabolic outcomes were defined as follows: (i) central obesity: waist circumference (WC) ≥90 cm in men, and WC ≥80 cm in women, in line with the Asian standard; (ii) raised blood pressure: systolic blood pressure ≥ 130 mmHg, diastolic blood pressure ≥ 85 mmHg, or pharmacologic treatment for hypertension; (iii) raised fasting serum glucose: fasting serum glucose ≥100 mg/dL or pharmacologic treatment for diabetes; (iv) elevated TG levels: serum TG ≥150 mg/dL or pharmacologic treatment for dyslipidemia; and (v) decreased HDL-C: levels of < 40 mg/dL in men and < 50 mg/dL in women or pharmacologic treatment for dyslipidemia. The presence of three or more is defined as metabolic syndrome.

Blood pressure was measured following the standard protocol using a mercury manometer [19]. Serum glucose, TG, and HDL-C levels were measured on a Hitachi 7600–210 automatic analyzer (Hitachi, Japan), using the hexokinase UV, enzymatic, and homogeneous enzymatic colorimetric methods, respectively.

### Covariates

We considered age, education, household income, smoking, alcohol drinking, and physical activity as covariates. Workers were defined as a paid working group to reduce the heterogeneity of work characteristics. We classified education into three categories based on the highest level of education as follows: (1) below middle school, (2) high school, and (3) university. Household income was divided into four quartiles. Smoking status was divided into three categories (current, former, and never). Alcohol intake was classified into three categories (severe, moderate, and none). Physical activity was defined as “yes” if the participant performed vigorous-intensity physical activity for at least 75 min, moderate-intensity physical activity for at least 150 min, or an equivalent combination of moderate and vigorous activity per week [20].

### Statistical analyses

All statistical analyses were performed using Statistical Analysis System version 9.4 (SAS Institute, Cary, NC, USA). Chi-square tests were conducted to assess the differences in general characteristics based on regular breakfast intake. Student t-tests were conducted to compare the average of each measurement of metabolic outcomes and the total number of abnormal metabolic outcomes, based on regular breakfast intake. Weighted prevalence of number of metabolic outcomes by breakfast consumption were calculated using the KNHANES sample weights which were constructed for sample participants to represent the Korean population by accounting for the complex survey design, survey non-response, and post-stratification. Number of abnormal metabolic outcomes showed non-normal distributions in the current study. The negative binomial model was selected to appropriate regression model considering Akaike information criteria and Bayesian information criteria, which are the criteria used for assessing model goodness of fit compared with Poisson or zero-inflated negative binominal model. Multivariate negative binominal regressions were used to explore the association between the risk of increasing number of abnormal metabolic outcomes and regularity of breakfast intake, with adjustments for age, education level, income level, smoking, alcohol intake, and physical activity according to sex, work status, and age stratification, to estimate the odds ratio (OR) and 95% confidence intervals (CIs).

## Results

The baseline characteristics according to regular breakfast intake are presented in Table 1. The total number of participants was 21,193 (9022 men and 12,171 women), and 11,952 (56.4%) were regular breakfast eaters. No significant difference was observed in regular breakfast intake between men and women. The participants were stratified based on age as young (age, 20–39 years) and middle-aged (age, 40–59 years). Among regular breakfast eaters, the percentage of young participants (*n* = 2775, 23.2%) was significantly lower than that of middle-aged participants (*n* = 9177, 76.8%). The proportion of participants with a high level of education was higher among irregular breakfast eaters. In contrast, the proportion of participants with a larger household income was higher among regular breakfast eaters.

**Table 1:** General characteristics of study participants by breakfast consumption

Characteristics	Regular breakfast, n (%)		*p*-value
	Yes	No	
Total	11,952 (56.4)	9241 (43.6)	
Sex			0.1100
Men	5031 (42.1)	3991 (43.2)	
Women	6921 (57.9)	5250 (56.8)	
Age			<.0001
20–39	2775 (23.2)	4544 (49.2)	
40–59	9177 (76.8)	4697 (50.8)	
Education			<.0001
Middle school	3671 (30.7)	1707 (18.5)	
High school	3878 (32.5)	3435 (37.2)	
College or more	4403 (36.8)	4099 (44.4)	
Household income			<.0001
1st Quartile	942 (7.9)	821 (8.9)	
2nd Quartile	2707 (22.7)	2278 (24.6)	
3rd Quartile	3785 (31.7)	3086 (33.4)	
4th Quartile	4518 (37.8)	3056 (33.1)	
Smoking			<.0001
None	8636 (72.3)	5856 (63.4)	
Past	1754 (14.7)	1304 (14.1)	
Current	1562 (13.1)	2081 (22.5)	
Drinking			<.0001
None	4122 (34.6)	2105 (22.8)	
Moderate	6743 (56.5)	5829 (63.1)	
Severe	1062 (8.9)	1300 (14.1)	
Physical activity			0.0008
No or unknown	7304 (61.1)	5438 (58.9)	
Yes	4648 (38.9)	3803 (41.2)	
Working			<.0001
No	7042 (58.9)	4754 (51.4)	
Yes	4910 (41.1)	4487 (48.6)	
Abdominal obesity			0.4420
No	9601 (80.3)	7384 (79.9)	
Yes	2351 (19.7)	1857 (20.1)	
Raised blood pressure			<.0001
No	8954 (74.9)	7305 (79.1)	
Yes	2998 (25.1)	1936 (20.9)	
Raised fasting glucose			<.0001
No	8932 (74.7)	7128 (77.1)	
Yes	3020 (25.3)	2113 (22.9)	
Increased TG			0.0195
No	8674 (72.6)	6839 (74.1)	
Yes	3278 (27.4)	2402 (26.9)	
Decreased HDL-C			<.0001
No	7877 (65.9)	6428 (69.6)	
Yes	4075 (34.1)	2813 (30.4)	
Metabolic syndrome			<.0001
No	9523 (79.7)	7617 (82.4)	
Yes	2429 (20.3)	1624 (17.6)	

Abdominal obesity: WC > 90 cm for male, WC > 80 cm for female (Asian modified)

Raised blood pressure: Systolic blood pressure ≥ 130 mmHg, Diastolic blood pressure ≥ 85 mmHg, or pharmacologic treatment for hypertension

Raised fasting plasma glucose: Plasma fasting glucose ≥ 100 mg/dL or pharmacologic treatment for diabetes

Increased triglyceride(TG): Plasma triglyceride ≥ 150 mg/dL or pharmacologic treatment for dyslipidemia

Decreased high density lipoprotein cholesterol(HDL-C): Plasma HDL-C < 40 mg/dL for male, Plasma-HDL-C < 50 mg/dL for female or pharmacologic treatment for dyslipidemia

Metabolic syndrome: Three or more of the above traits (NCEP-ATP III Criteria)

The percentage of those with unhealthy lifestyle habits was higher among irregular breakfast eaters, except for physical activity. In our study, 13.1% of regular breakfast eaters, compared with 22.5% of irregular breakfast eaters, were current smokers. Furthermore, only 8.9% of regular breakfast eaters were heavy drinkers, compared with 14.1% of the irregular breakfast eaters. There was no significant difference in physical activity level between regular breakfast and irregular breakfast eaters. Individuals with abnormal metabolic outcomes, except abdominal obesity, significantly tend to demonstrate regular breakfast consumption habits. The specific metabolic outcomes stratified by working population are shown in Table 2. We considered the average value of each metabolic outcome according to the breakfast consumption status. Age was not stratified in the above analysis.

**Table 2:** Metabolic outcomes by breakfast consumption

Characteristics	Regular breakfast, mean (standard deviation)		p-value
	Yes	No	
Total participants			
Men			
Abdominal circumference (cm)	82.53 (11.2)	83.83 (10.8)	0.0083
Systolic blood pressure (mmHg)	116.7 (13.7)	116.8 (12.7)	0.9456
Diastolic blood pressure (mmHg)	76.32 (11.5)	77.53 (10.9)	<.0001
Fasting glucose level (mg/dL)	99.74 (22.5)	98.05 (21.2)	0.0003
TG level (mg/dL)	149.6 (13.7)	156.8 (13.8)	0.0128
HDL-C level (mg/dL)	48.14 (10.9)	47.95 (10.9)	0.3939
Number of abnormal metabolic outcomes	1.52 (1.5)	1.51 (1.4)	0.7491
Women			
Abdominal circumference (cm)	75.69 (9.6)	75.30 (9.7)	0.0285
Systolic blood pressure (mmHg)	111.2 (14.8)	109.0 (13.3)	<.0001
Diastolic blood pressure (mmHg)	72.47 (9.9)	72.15 (9.5)	0.0662
Fasting glucose level (mg/dL)	94.78 (18.1)	93.68 (18.2)	0.0010
TG level (mg/dL)	105.9 (79.5)	101.7 (73.9)	0.0028
HDL-C level (mg/dL)	55.06 (12.2)	56.30 (12.3)	<.0001
Number of abnormal metabolic outcomes	1.17 (1.3)	0.97 (1.2)	<.0001
Non-working population			
Men			
Abdominal circumference (cm)	80.18 (12.5)	81.96 (11.8)	<.0001
Systolic blood pressure (mmHg)	115.4 (13.5)	116.2 (13.0)	0.0306
Diastolic blood pressure (mmHg)	73.47 (12.2)	75.61 (11.4)	<.0001
Fasting glucose level (mg/dL)	98.98 (21.6)	97.84 (22.2)	0.0841
TG level (mg/dL)	136.7 (12.5)	148.0 (13.5)	0.0042
HDL-C level (mg/dL)	48.47 (10.9)	48.45 (10.8)	0.9474
Number of abnormal metabolic outcomes	1.34 (1.4)	1.36 (1.4)	0.6741
Women			
Abdominal circumference (cm)	75.19 (10.0)	75.41 (10.2)	0.3559
Systolic blood pressure (mmHg)	110.9 (14.7)	109.0 (13.3)	<.0001
Diastolic blood pressure (mmHg)	71.89 (10.1)	71.87 (9.6)	0.9338
Fasting glucose level (mg/dL)	94.58 (17.5)	94.20 (20.1)	0.4166
TG level (mg/dL)	107.7 (77.0)	104.2 (72.1)	0.0500
HDL-C level (mg/dL)	54.51 (11.9)	55.68 (12.3)	<.0001
Number of abnormal metabolic outcomes	1.18 (1.3)	1.03 (1.2)	<.0001
Working population			
Men			
Abdominal circumference (cm)	85.27 (8.7)	85.40 (9.5)	0.6219
Systolic blood pressure (mmHg)	118.3 (13.8)	117.2 (12.4)	0.0035
Diastolic blood pressure (mmHg)	79.65 (9.8)	79.14 (10.2)	0.0925
Fasting glucose level (mg/dL)	100.6 (23.5)	98.23 (20.4)	0.0003
TG level (mg/dL)	164.7 (14.8)	164.3 (14.0)	0.9257
HDL-C level (mg/dL)	47.77 (10.8)	47.53 (10.9)	0.4624
Number of abnormal metabolic outcomes	1.72 (1.4)	1.63 (1.4)	0.0375
Women			
Abdominal circumference (cm)	76.53 (8.9)	75.16 (9.1)	<.0001
Systolic blood pressure (mmHg)	111.7 (14.9)	108.9 (13.2)	<.0001
Diastolic blood pressure (mmHg)	73.45 (9.6)	72.49 (9.3)	0.0004
Fasting glucose level (mg/dL)	95.12 (19.1)	93.02 (15.4)	<.0001
TG level (mg/dL)	102.9 (83.5)	98.56 (76.0)	0.0580
HDL-C level (mg/dL)	56.00 (12.4)	57.09 (12.4)	0.0021
Number of abnormal metabolic outcomes	1.16 (1.3)	0.91 (1.1)	<.0001

*TG* Triglyceride

*HDL-C* High density lipoprotein - Cholesterol

The weighted prevalence of abnormal metabolic outcome according to breakfast consumption status, stratified by age, sex, and working status is presented in Table 3. After stratification of sex, age, and working status, there was a significant difference in weighted prevalence between regular and irregular breakfast eaters. Among young male workers, 41.0 and 34.9% of regular and irregular breakfast eaters, respectively, had normal metabolic outcomes, with zero metabolic abnormality. In contrast, in the middle-aged female worker group, regular breakfast eaters had a significantly higher number of metabolic abnormalities.

**Table 3:** Weighted prevalence of abnormal metabolic outcome by breakfast consumption

	Number of metabolic abnormalities, Regular / Irregular breakfast, % of row (standard errors)						p-value
	0	1	2	3	4	5	
Total participants (n = 27,627,602)	37.3 (0.4)/ 40.7 (0.3)	25.6 (0.3)/ 25.8 (0.3)	16.8 (0.2)/ 15.9 (0.2)	11.0 (0.2)/ 10.1 (0.2)	6.9 (0.1)/ 5.3 (0.1)	2.3 (0.1)/ 2.1 (0.1)	<.0001
Men (*n* = 13,597,358)	32.6 (0.5)/ 33.0 (0.5)	22.9 (0.4)/ 24.2 (0.3)	18.9 (0.3)/ 18.7 (0.3)	13.4 (0.3)/ 13.5 (0.3)	9.0 (0.2)/ 7.6 (0.2)	3.2 (0.1)/ 3.0 (0.1)	0.3096
Younger aged Men (n = 5,534,982)	42.2 (0.8)/ 36.1 (0.8)	25.4 (0.6)/ 26.0 (0.7)	17.6 (0.5)/ 18.0 (0.7)	8.7 (0.3)/ 11.6 (0.5)	5.1 (0.3)/ 6.3 (0.4)	1.0 (0.1)/ 2.0 (0.2)	0.0067
Middle aged Men (*n* = 8,062,377)	29.1 (0.6)/ 29.1 (0.5)	22.0 (0.5)/ 21.8 (0.4)	19.4 (0.5)/ 19.6 (0.4)	15.1 (0.4)/ 15.9 (0.4)	10.4 (0.4)/ 9.2 (0.3)	4.0 (0.2)/ 4.3 (0.2)	0.8014
Women (*n* = 14,030,244)	41.8 (0.5)/ 48.5 (0.5)	28.2 (0.4)/ 27.5 (0.4)	14.8 (0.3)/ 13.0 (0.3)	8.8 (0.2)/ 6.6 (0.2)	5.1 (0.2)/ 3.1 (0.1)	1.5 (0.1)/ 1.2 (0.1)	<.0001
Younger aged Women (n = 5,408,303)	58.1 (0.7) / 57.4 (0.9)	26.6 (0.5) / 26.3 (0.6)	10.3 (0.3) / 10.6 (0.4)	3.1 (0.2) / 3.9 (0.3)	1.3 (0.1) / 1.3 (0.1)	0.6 (0.1) / 0.5 (0.1)	0.8674
Middle aged Women (n = 8,621,941)	35.8 (0.6) / 38.7 (0.5)	28.7 (0.5) / 28.9 (0.4)	16.4 (0.4) / 15.7 (0.3)	10.9 (0.3) / 9.6 (0.2)	6.4 (0.2) / 5.0 (0.2)	1.8 (0.1) / 2.0 (0.1)	0.0516
Non-working population (n = 14,787,523)	39.6 (0.5) / 42.6 (0.4)	26.1 (0.4) / 25.6 (0.3)	15.3 (0.3) / 15.1 (0.3)	10.3 (0.2) / 9.2 (0.2)	6.6 (0.2) / 5.3 (0.2)	2.1 (0.1) / 2.2 (0.1)	0.0165
Men (*n* = 6,443,456)	37.4 (0.7) / 37.3 (0.7)	22.8 (0.6) / 23.6 (0.5)	15.6 (0.5) / 17.4 (0.5)	12.5 (0.4) / 11.7 (0.4)	8.5 (0.3) / 7.1 (0.3)	3.1 (0.2) / 3.0 (0.2)	0.4675
Younger aged Men (n = 1,990,178)	44.1 (1.3) / 38.1 (1.5)	26.8 (1.0) / 26.8 (1.2)	14.9 (0.8) / 17.0 (1.1)	8.3 (0.5) / 10.6 (0.8)	5.5 (0.5) / 5.6 (0.7)	0.4 (0.1) / 1.9 (0.3)	0.2059
Middle aged Men (n = 4,453,278)	35.8 (0.8) / 36.6 (0.7)	21.9 (0.7) / 20.9 (0.5)	15.9 (0.6) / 17.7 (0.5)	13.5 (0.6) / 12.6 (0.4)	9.3 (0.5) / 8.3 (0.3)	3.7 (0.3) / 3.9 (0.2)	0.7652
Women (n = 8,344,068)	41.2 (0.6) / 46.8 (0.6)	28.6 (0.5) / 27.2 (0.5)	15.1 (0.4) / 13.2 (0.3)	8.7 (0.3) / 7.2 (0.2)	5.1 (0.2) / 3.9 (0.2)	1.4 (0.1) / 1.6 (0.1)	0.0003
Younger aged Women (n = 2,754,380)	55.9 (1.0) / 56.0 (1.1)	26.9 (0.7) / 25.3 (0.9)	11.8 (0.5) / 11.3 (0.6)	3.1 (0.2) / 5.0 (0.4)	1.5 (0.1) / 1.8 (0.2)	0.9 (0.2) / 0.6 (0.2)	0.4899
Middle aged Women (n = 5,589,687)	36.9 (0.7) / 38.5 (0.6)	29.1 (0.6) / 29.0 (0.5)	16.1 (0.5) / 15.0 (0.4)	10.2 (0.4) / 9.3 (0.3)	6.1 (0.3) / 5.8 (0.2)	1.5 (0.1) / 2.4 (0.1)	0.2629
Working population (*n* = 12,840,079)	34.4 (0.5) / 38.9 (0.5)	25.0 (0.4) / 26.1 (0.4)	18.7 (0.3) / 16.6 (0.3)	11.9 (0.3) / 10.9 (0.3)	7.4 (0.2) / 5.4 (0.2)	2.6 (0.1) / 2.1 (0.1)	<.0001
Men (*n* = 7,153,902)	27.6 (0.6) / 29.7 (0.6)	23.0 (0.5) / 24.6 (0.6)	22.2 (0.5) / 19.7 (0.5)	14.3 (0.4) / 14.9 (0.5)	9.4 (0.3) / 8.0 (0.3)	3.4 (0.2) / 3.0 (0.2)	0.1410
Younger aged Men (*n* = 3,544,804)	41.0 (0.9) / 34.9 (1.0)	24.6 (0.7) / 25.5 (1.0)	19.2 (0.6) / 18.5 (0.8)	8.9 (0.4) / 12.2 (0.7)	4.8 (0.3) / 6.7 (0.5)	1.4 (0.2) / 2.1 (0.3)	0.0458
Middle aged Men (n = 3,609,098)	20.8 (0.8) / 20.0 (0.6)	22.2 (0.8) / 23.0 (0.6)	23.8 (0.8) / 21.8 (0.6)	17.1 (0.7) / 20.0 (0.6)	11.8 (0.6) / 10.3 (0.6)	4.4 (0.4) / 4.9 (0.3)	0.4969
Women (n = 5,686,176)	42.8 (0.7) / 50.5 (0.8)	27.5 (0.6) / 27.9 (0.6)	14.2 (0.4) / 12.8 (0.4)	9.0 (0.3) / 5.9 (0.3)	5.0 (0.2) / 2.1 (0.2)	1.6 (0.1) / 0.8 (0.1)	<.0001
Younger aged Women (n = 2,653,923)	60.5 (1.0) / 58.8 (1.3)	26.3 (0.7) / 27.2 (0.9)	8.7 (0.4) / 9.9 (0.6)	3.1 (0.2) / 2.9 (0.3)	1.2 (0.1) / 0.8 (0.2)	0.3 (0.1) / 0.4 (0.1)	0.8927
Middle aged Women (n = 3,032,254)	33.5 (0.8) / 38.9 (0.8)	28.1 (0.8) / 28.8 (0.7)	17.1 (0.6) / 16.8 (0.5)	12.1 (0.5) / 10.2 (0.4)	7.0 (0.4) / 3.9 (0.3)	2.3 (0.2) / 1.4 (0.1)	0.0033

The association between metabolic abnormalities and irregular breakfast consumption after adjusting for age, education, household income, smoking status, alcohol drinking status, and physical activity is presented in Table 4. Negative binomial regression analysis revealed that an irregular breakfast group had a higher risk of increased number of metabolic abnormalities in the younger working men population than regular breakfast group (odds ratio, 1.15; 95% confidence interval, 1.03–1.27). There was no significant association between the number of metabolic abnormalities and irregular breakfast consumption in middle-aged female workers after adjustment.

**Table 4:** Association between the number of metabolic abnormalities and irregular breakfast consumption, using negative binomial regression

	Odds Ratio (95% Confidence Interval) referred regular breakfast group in each category
Total participants (*n* = 21,193)	**1.05 (1.01–1.08)**
Men (*n* = 9022)	**1.11 (1.06–1.15)**
Younger aged Men (*n* = 3065)	**1.14 (1.06–1.23)**
Middle aged Men (*n* = 5957)	**1.08 (1.03–1.14)**
Women (n = 12,171)	1.02 (0.98–1.07)
Younger aged Women (*n* = 4254)	1.06 (0.96–1.17)
Middle aged Women (*n* = 7917)	1.01 (0.96–1.07)
Non-working population (*n* = 11,796)	1.05 (1.00–1.10)
Men (*n* = 4527)	**1.07 (1.01–1.14)**
Younger aged Men (*n* = 1066)	1.12 (0.97–1.29)
Middle aged Men (*n* = 3461)	1.04 (0.97–1.12)
Women (*n* = 7269)	1.06 (1.00–1.12)
Younger aged Women (*n* = 2177)	1.09 (0.96–1.25)
Middle aged Women (*n* = 5092)	1.06 (0.99–1.13)
Working population (*n* = 9397)	1.05 (1.00–1.10)
Men (*n* = 4495)	**1.14 (1.08–1.21)**
Younger aged Men (*n* = 1999)	**1.15 (1.03–1.27)**
Middle aged Men (*n* = 2496)	**1.12 (1.04–1.20)**
Women (*n* = 4902)	0.99 (0.92–1.06)
Younger aged Women (*n* = 2077)	1.03 (0.90–1.18)
Middle aged Women (*n* = 2825)	0.96 (0.89–1.05)

All models are adjusted for age, educational level, income level, smoking, alcohol drinking, and physical activity

Bolds are indicated statistical significance. (p-value < 0.05)

Younger population is 20–39 years old

Middle-aged population is 40–59 years old

## Discussion

We conducted this study to understand the association between regular breakfast intake and metabolic outcomes by sex, work status, and age group stratification. The breakfast consumption pattern was consistent with previous studies. Of the total participants, 56.4% consumed breakfast regularly. Similar patterns was observed in a previous Korean study using KNHANES 2017 data, 57.9% [21].

The results of this study showed that irregular breakfast intake (< 5 times per week) was closely linked to higher risk of increasing number of abnormal metabolic outcomes, especially in young men in the working population than regular breakfast intake. These results are consistent with a previous study that used the KNHANES data and showed that breakfast consumption patterns were associated with a risk of metabolic outcomes [22]. Furthermore, our results corresponds with that of a review article, which reported that daily breakfast consumers were less likely to have cardiovascular disease risk factors, including elevated serum LDL-C levels, low serum HDL-C levels, and elevated blood pressure [23].

As shown in Tables 1 and 2, regular breakfast eaters had more abnormal metabolic outcomes. After stratification by age, sex, and working status, young male workers and middle-aged female workers had significant differences. Table 3 shows that regular breakfast eaters among young male workers tended to have a smaller number of metabolic abnormalities, while regular breakfast eaters in the middle-aged female worker group had a larger number of metabolic abnormalities. However, after adjusting for covariates, the significance disappeared only in the middle-aged female worker group.

According to a previous study, there was no significant association between breakfast skipping and abnormal metabolic outcomes in women. A Japanese longitudinal cohort study on factory employees showed that the average frequency of breakfast skipping was not associated with BMI and waist circumference in women [24]. Our results are consistent with those of the aforementioned study, and there are several explanations for this result. Postmenopausal status is known to be associated with abnormal metabolic outcomes. In middle-aged women, postmenopausal status has been reported to affect the outcome [25]. One study reported lower BMI and appearance-related satisfaction levels among young Korean university female students compared with European and American students [26]. This could increase the risk of eating disorders in young women, which might have affected the results.

Another cross-sectional study using the KNHANES data reported a different result and stated that the risk of abnormal metabolic outcomes increased in both men and women [27]. However, the definition of breakfast skipping in that study was different from that of our study. In that study, a breakfast skipper was defined as a subject who had skipped breakfast 1 day or 2 days before the survey. This definition has a limitation in the overall representation of breakfast consumption. From 2013, KNHANES changed the question regarding breakfast consumption habit from “Did you skip breakfast before 1 day or 2 days?” to “What is the average frequency of breakfast consumption per week for the past 1 year?” We considered the latter question in this study.

Herein, we have proposed several mechanisms to explain the association between abnormal metabolic outcomes and breakfast skipping. Breakfast is the very first meal of the day, which kick-starts the daily metabolism of the human body. Energy consumption will be lower than the energy requirement if breakfast is skipped before going to work. Food deprivation is known to cause a reduction in the basal metabolic rate (BMR) via compensatory metabolism [28]. The reduction in the BMR leads to the consumption of excess calories, ultimately leading to weight gain.

The time of meal consumption affects the postprandial increase in energy expenditure and blood glucose levels. A randomized repeated-measures study showed that skipping breakfast was compensated by consuming big meals at lunch. In addition, the study found that breakfast skipping increased the overall 24 h average blood glucose levels [29]. Another study found that breakfast skipping was associated with higher hemoglobin A1c values, which indicate poorer glycemic control [30]. A longitudinal study showed that breakfast skippers had high levels of fasting insulin [31]. Poor glycemic control is associated with high levels of glucose, insulin resistance, and high levels of fasting insulin. Insulin is known to stimulate hydroxy-methyl-glutaryl Co-A reductase activity, which plays a crucial role in the biosynthesis of cholesterol and lipids. Through these mechanisms, breakfast skipping might lead to increased fasting glucose levels, increased blood pressure, high levels of serum TG, and low levels of HDL-C.

This study observed a more significant relationship between breakfast skipping and abnormal metabolic outcomes in men in the working group than in women in all other groups. A previous study indicated that men in the working group, compared with women in the same group, had a higher risk of metabolic syndrome associated with working conditions [32]. Another study reported a significantly increased risk of metabolic syndrome in working men compared with working women [33]. The results of this study further support the idea of the working male population being vulnerable to metabolic diseases.

To the best of our knowledge, our study is the first and largest sample-sized study to explore the association between abnormal metabolic outcomes and breakfast skipping in the Korean population. Only a few studies have investigated the effect of work status on the association between breakfast skipping and abnormal metabolic outcomes. Our research indicated that the detrimental effect of breakfast skipping was evident in the working Korean male population, especially in young adults. Educating young male workers regarding the benefits of eating breakfast could be a great way to prevent further metabolic diseases.

This study identified the relationship between breakfast skipping and the number of metabolic abnormalities and proposed a novel hypothesis to explain the variable strength of association according to the stratifications. We considered stratifications, such as age and work status, which had not been used in previous studies. Work status is an important factor that affects daily metabolism. The different strengths of association according to work status implies that daily activity or stress levels might be an effect modifier of the association between breakfast skipping and abnormal metabolic outcomes.

Our study has several limitations. First, we used a self-administered questionnaire to acquire information about breakfast consumption. This study used a self-reported questionnaire for breakfast consumption because the use of a self-reported questionnaire is common in breakfast consumption studies, and its reliability has been clinically verified in highly cited and qualified studies [3, 6]. Moreover, the proportion of regular breakfast eaters in this study was similar to that of previous study [21]. This shows the repeatability of the questionnaire. The large sample size in our study could also reduce the effect of the error. In addition, our questionnaire was designed to include the 1-year average frequency to appropriately reflect the long-term dietary habits of the participants.

Second, considering the cross-sectional design of our study, caution must be exercised to establish a causal relationship. A longitudinal interventional study is needed to definitively unveil the exact mechanism. Third, although we stratified participants based on work status, we did not examine specific working conditions such as shift work, long working hours, manual work, and clerical work. Further analysis based on working conditions is required to determine whether breakfast skipping is an important risk factor for abnormal metabolic outcomes in the working population.

Finally, since the energy requirement for work was not quantified in this study, we could not directly compare the morning energy expenditure between the working and non-working populations. Further detailed studies are required to reveal the relationship between early morning working, breakfast skipping, and the risk of abnormal metabolic outcomes. Previous studies reported the significant association between skipping breakfast and diet quality [34, 35]. Due to the lack of data on dietary quality, the quality and quantity of nutrients could not be analyzed in this study. The quality and quantity of nutrients in relation to breakfast skipping need to be clarified in future studies.

Although breakfast is considered the most important meal of the day, the percentage of regular breakfast eaters among young adults was only 37.92%. This trend is in progress, accelerating the risk of metabolic outcomes among young adults. The risk is accentuated in the working population of young men, and further studies are required to clarify the association between specific working conditions (working hours or shift work), breakfast habituation, and the risk of metabolic outcomes.

## Conclusion

Our study showed that breakfast skipping is associated with abnormal metabolic outcomes in the Korean male population, especially in young workers, and provided novel ideas to explain the mechanism through which breakfast skipping affects metabolic outcomes.

## Data Availability

KNHANES data are publicly available. (https://knhanes.cdc.go.kr/knhanes/eng/index.do).

## Abbreviations

KNHANES: Korea National Health and Nutrition Examination Survey
BMI: Body mass index
LDL-C: Low-density lipoprotein cholesterol
HDL-C: High-density lipoprotein cholesterol
TG: Triglyceride
BMR: Basal metabolic rate
